# Evidence for Involvement of the *Salmonella enterica* Z-Ring Assembly Factors ZapA and ZapB in Resistance to Bile

**DOI:** 10.3389/fmicb.2021.647305

**Published:** 2021-02-25

**Authors:** Rocío Fernández-Fernández, Sara B. Hernández, Elena Puerta-Fernández, María A. Sánchez-Romero, Verónica Urdaneta, Josep Casadesús

**Affiliations:** Departamento de Genética, Facultad de Biología, Universidad de Sevilla, Sevilla, Spain

**Keywords:** *Salmonella*, bile resistance, Z-ring, Lon protease, MicA sRNA

## Abstract

Genes annotated as *ygfE* and *yiiU* in the genome of *Salmonella enterica* serovar Typhimurium encode proteins homologous to *Escherichia coli* cell division factors ZapA and ZapB, respectively. ZapA^−^ and ZapB^−^ mutants of *S. enterica* are bile-sensitive. The amount of *zapB* mRNA increases in the presence of a sublethal concentration of sodium deoxycholate (DOC) while *zapA* mRNA remains unaffected. Increased *zapB* mRNA level in the presence of DOC is not caused by upregulation of *zapB* transcription but by increased stability of *zapB* mRNA. This increase is suppressed by an *hfq* mutation, suggesting the involvement of a small regulatory RNA. We provide evidence that such sRNA is MicA. The ZapB protein is degraded in the presence of DOC, and degradation appears to involve the Lon protease. We propose that increased stability of *zapB* mRNA in the presence of DOC may counter degradation of bile-damaged ZapB, thereby providing sufficient level of functional ZapB protein to permit Z-ring assembly in the presence of bile.

## Introduction

During infection of the gastrointestinal tract, enteric pathogens must endure harsh environments such as pH variations, low oxygen levels, elevated osmolarity, and nutrient limitation (Alvarez-Ordonez et al., 2011; Fang et al., 2016). In the small intestine, periodical secretion of bile poses another challenge due to the antibacterial activity of bile salts (Hofmann and Hagey, 2008). *Salmonella* serovars that cause systemic and chronic infections also encounter bile in the gall bladder, at concentrations higher and more steady than in the intestine (Hofmann and Hagey, 2008; Dougan and Baker, 2014). Bile acids/salts act as detergents that disrupt membrane phospholipids, cause misfolding and denaturation of proteins as well as oxidative DNA damage, and interfere with the formation of secondary RNA structures (Bernstein et al., 1999; Gunn, 2000; Prieto et al., 2004; Begley et al., 2005; Merritt and Donaldson, 2009; Urdaneta and Casadesus, 2017; Bustos et al., 2018).

Resistance to bile can be studied under laboratory conditions by adding ox bile or individual bile salts to microbiological culture media. In both *Escherichia coli* and *Salmonella*, isolation of bile-sensitive mutants has identified loci required for bile resistance, and suppressor analysis has contributed to understand the mechanisms involved (Picken and Beacham, 1977; Thanassi et al., 1997; van Velkinburgh and Gunn, 1999; Wibbenmeyer et al., 2002; Ramos-Morales et al., 2003; Buckley et al., 2006; Crawford et al., 2012; Hernandez et al., 2012; May and Groisman, 2014; Hernandez et al., 2015; Urdaneta and Casadesus, 2018). In the last decade, bacterial responses to bile have been investigated also by high throughput analysis of gene expression, and the combination of genetics, biochemistry, and transcriptomics has provided a picture of the cell components and bacterial responses that permit bile resistance (Prouty et al., 2004; Hernandez et al., 2012; Kroger et al., 2013; Johnson et al., 2017). Envelope structures such as the lipopolysaccharide (Picken and Beacham, 1977; van Velkinburgh and Gunn, 1999; Crawford et al., 2012), the enterobacterial common antigen (Ramos-Morales et al., 2003), and the peptidoglycan (Hernandez et al., 2015) provide barriers that reduce intake of bile salts. In addition, bile salts are transported outside the cell by efflux pumps, especially AcrAB-TolC (Thanassi et al., 1997; Buckley et al., 2006; Urdaneta and Casadesus, 2018). Exposure to bile also activates stress responses (Thanassi et al., 1997; Bernstein et al., 1999; Hernandez et al., 2012), remodeling of the cell wall (Hernandez et al., 2015), and DNA repair (Prieto et al., 2006).

Among the bile-upregulated genes identified by transcriptomic analysis, a *Salmonella enterica* locus annotated as *yiiU* was found (Hernandez et al., 2012). In this study, we show that *S. enterica* YiiU is a homolog of the *E. coli* ZapB protein, a non-essential cell division factor involved in Z-ring formation and nucleoid segregation (Ebersbach et al., 2008; Buss et al., 2015; Mannik et al., 2016; Du and Lutkenhaus, 2017). *Salmonella* ZapB^−^ mutants are sensitive to sodium deoxycholate (DOC), indicating that ZapB plays a role in bile resistance. We also show that the *S. enterica ygfE* gene is a homolog of *E. coli* ZapA, and that ZapA^−^ mutants are sensitive to bile. However, *zapA* expression is not regulated by bile.

Upregulation of the *zapB* mRNA level in the presence of DOC appears to involve interaction with MicA, a small regulatory RNA. We tentatively propose that increased stability of *zapB* mRNA in the presence of DOC may counter degradation of bile-damaged ZapB by the Lon protease.

## Materials and Methods

### Bacterial Strains, Bacteriophages, Plasmids, and Strain Construction

Strains of *S. enterica* used in this study (Table 1) belong to serovar Typhimurium and derive from a His^+^ derivative of SL1344 (Hoiseth and Stocker, 1981; Cano et al., 2002). *Escherichia coli* RK4353 [*Δ(argF-lac)169 Δ(fimB-fimE)632(::IS1) Δ(fruK-yeiR) F^−^ λ^−^ [araD139] deoC1 flhD5301 gyrA219 rbsR22 relA1 rpsL150*] was the host of pBAD18 (Guzman et al., 1995). *Escherichia coli* DH5α [*endA1 hsdR17 supE44 thi1 recA1 gyrA96 relA1 ΔlacU189* (Ф80 *lacZΔM15*)] was also used for cloning. *E. coli* CC118 λ *pir* [*phoA20 thi-1 rspE rpoB argE(Am) recA1* (λ *pir*)] and *E. coli* S17-1 λ *pir* [*recA pro hsdR* RP4-2-Tc::Mu-Km::Tn7 (λ *pir*)] were used for directed construction of point mutations. Plasmid pDMS197 is a suicide vector containing the *sacB* gene of *Bacillus* spp. (Edwards et al., 1998). Transduction was performed with *S. enterica* phage P22 HT 105/1 *int201* (Schmieger, 1972). The P22 HT transduction protocol was described elsewhere (Torreblanca et al., 1999). To obtain phage-free isolates, transductants were purified by streaking on green plates (Garzon et al., 1995). Phage sensitivity was tested by cross-streaking with P22 H5.

**Table 1:** Strains of *Salmonella enterica* serovar Typhimurium.

Strain	Genotype
SV5370	*hfq::* Cm^R^
SV6268	*zapB::*Km^R^
SV6269	*∆zapB*
SV6270	*zapB::lacZ*^a^
SV6340	*∆zapB asmA::* Km^R^
SV6898	*zapB::*3 × FLAG
SV7016	*lon*::Tn*10*
SV7017	*zapB::*3 × FLAG *lon::*Tn*10*
SV7022	*∆zapB lon::*Tn*10*
SV7264	SL1344/pBAD18
SV7265	*∆zapB*/pBAD18
SV7267	SL1344/pBAD18-*zapB*
SV7268	*∆zapB*/pBAD18-*zapB*
SV7300	*zapA::*Km^R^
SV7338	SL1344/pIC552
SV7339	SL1344/pIC552-p*_zapB_::lac*
SV7429	pL_tetO_::*zapB::lacZ*
SV7432	*∆zapB*/pBAD18-*zapB Escherichia coli*
SV9265	*zapB* ΔCDS (coding sequence deleted)
SV9371	*zapB* + 9 nt::*lacZ*
SV9372	*zapB* + 18 nt::*lacZ*
SV9373	*zapB* + 27 nt::*lacZ*
SV9374	*zapB* + 30 nt::*lacZ*
SV9432	*zapB::lacZ* ∆3'UTR
SV9439	*zapB* + 18::*lacZ*/pBR328
SV9442	*zapB* + 18::*lacZ*/pBR328-MicA
SV9443	*zapB::lacZ*/pBR328-MicA
SV9445	*zapB* + 18::*lacZ*/pBR328-InvR
SV9446	*zapB::lacZ*/pBR328-InvR
SV9448	*zapB* + 18::*lacZ*/pBR328-CyaR
SV9449	*zapB::lacZ*/pBR328-CyaR
SV9451	*zapB::lacZ*/RyhB-1
SV9451	*zapB* + 18::*lacZ*/pBR328-RyhB-1
SV9486	*zapB* + 18::*lacZ*/pBR328-MicA*
SV9647	zapB*50A-54 T-58A-61G
SV9770	*ΔzapA ΔzapB*
SV9771	SL1344/pBAD18-*zapA*
SV9772	*ΔzapA*/pBAD18
SV9773	*ΔzapA*/pBAD18-*zapA*
SV9774	*zapB::lacZ*/pBR328-*RyhB-2*
SV9775	*ΔzapA*/pBAD18::*zapA E. coli*

Targeted disruption of *yiiU* (*zapB*) and *ygfE* (*zapA*) was achieved using the pKD13 plasmid (Datsenko and Wanner, 2000) and oligonucleotides listed in Supplementary Table S1. The antibiotic resistance cassette introduced during strain construction was excised by recombination with pCP20 (Datsenko and Wanner, 2000). For construction of the *lacZ* fusions in the *Salmonella* chromosome, FRT sites generated by excision of the Km^R^ cassette were used to integrate plasmid pCE40 (Ellermeier et al., 2002). Addition of 3xFLAG tag to the protein-coding *zapB* DNA sequence was carried out using the pSUB11 plasmid as template (Uzzau et al., 2001) and the oligonucleotides zapB-FLAG-FOR and zapB-FLAG-REV. Introduction of the *lon*::Tn10 allele into SL1344 was achieved by P22 HT-mediated transduction from the ATCC 14028 derivative SV5663 (Garcia-Calderon et al., 2009). The P_LtetO_-*zapB* construct was engineered by inserting the P_LtetO_ promoter from the pXG1 plasmid (Lutz and Bujard, 1997) upstream of the transcriptional start site of *zapB::lacZ* on the *Salmonella* chromosome using primers zapB-PLtetO-FOR and zapB-PLtetO-REV. Details on the procedure have been described elsewhere (Lopez-Garrido and Casadesus, 2012).

To clone the *zapB* promoter with its 5' untranslated region (UTR) upstream of the *lacZ* gene of pIC552 (Macian et al., 1994), the primers pIC-zapB-FOR and pIC-zapB-REV were used. Plasmids carrying the *S. enterica* and *E. coli zapA* and *zapB* genes were constructed by cloning the respective genes under the control of the arabinose-dependent p_BAD_ promoter of pBAD18 (Guzman et al., 1995). Plasmids pBR328-CyaR, pBR328-InvR, pBR328-MicA, pBR328-RyhB-1, and pBR328-RyhB-2 are pBR328 derivatives.

### Media and Culture Conditions

Lysogeny broth (LB) and M9 minimal medium (Sambrook and Russell, 2001) were used as liquid media. Solid LB contained agar at 1.5% final concentration. To prepare LB containing sodium DOC (Sigma-Aldrich), an appropriate volume from a 25% stock was added. Liquid cultures were grown with aeration by shaking in an orbital incubator. Antibiotics were used at concentrations described previously (Garcia-Calderon et al., 2005). Arabinose was used at a final concentration of 0.2%.

### Subcellular Fractionation

Subcellular fractionation was performed as described elsewhere (Pucciarelli et al., 2002), with some modifications. Briefly, bacteria were grown in LB medium at 37°C, spun down by centrifugation at 15,000 × *g* for 5 min at 4°C and resuspended twice in cold phosphate-buffered saline (PBS, pH 7.4). The bacterial suspension was disrupted by sonication, and unbroken cells were further removed by low-speed centrifugation (5,000 × *g*, 5 min, 4°C). The supernatant was centrifuged at high speed (100,000 × *g*, 30 min, 4°C) and the new supernatant was recovered as the cytosol fraction. The pellet containing envelope material was suspended in PBS with 0.4% Triton X-100 and incubated for 2 h at 4°C. The sample was centrifuged again (100,000 × *g*, 30 min, 4°C) and divided into the supernatant containing mostly inner membrane proteins and the insoluble fraction corresponding to the outer membrane fraction. An appropriate volume of Laemmli buffer was added to each fraction. After heating (100°C, 5 min) and clearing by centrifugation (15,000 × *g*, 5 min, room temperature), the protein content was analyzed by SDS-PAGE.

### Determination of Minimal Inhibitory Concentration of Sodium Deoxycholate

Aliquots from exponential cultures in LB, each containing around 3 × 10^2^ colony-forming-units (CFUs) were transferred to polypropylene microtiter plates (Soria Genlab, Valdemoro, Spain) containing known amounts of DOC. After 12 h incubation at 37°C, growth was visually monitored.

### β-Galactosidase Assays

Levels of β-galactosidase activity were determined using the CHCl_3_-sodium dodecyl sulfate permeabilization procedure (Miller, 1972).

### Quantitative Reverse Transcriptase PCR

For quantitative reverse transcriptase-PCR (qRT-PCR), *Salmonella* RNA was extracted from late exponential cultures (OD_600_ ≅ 1). Gene-specific primers (Supplementary Table S1) were designed with ProbeFinder software^1^ from Roche Applied Science. The efficiency of each primer pair was determined to be higher than 90%, following the procedure described in the real-time quantitative PCR (qPCR) guide of Applied Biosystems. Relative RNA levels were determined using the ∆∆Ct method. Each ∆∆Ct determination was performed at least in three different RNA samples.

### Preparation of Protein Extracts and Western Blot Analysis

Total protein extracts were prepared from cultures grown until stationary phase (OD_600_ > 1) at 37°C in LB or LB + 5% DOC. Around 3 × 10^9^ bacterial cells were collected by centrifugation (16,000 × *g*, 2 min), resuspended in 50 μl of Laemmli sample buffer [1.3% SDS, 10% (v/v) glycerol, 50 mM Tris-HCl, 1.8% β-mercaptoethanol, 0.02% bromophenol blue, pH 6.8] and boiled (100°C, 5 min). Before loading, the samples were cleared by centrifugation (15,000 × *g*, 5 min). For analysis of protein stability, cultures were grown in LB at 37°C with shaking to a OD_600_ of 0.6–0.8. Chloramphenicol (40 μg ml^−1^) was added, and the culture was divided into two types: the DOC sample, to which 25% of DOC was added to reach a final concentration of 5%, and the LB sample, to which LB was added to equalize the volume with the DOC sample. A 1 ml aliquot was removed as zero-time control, and the samples were further incubated at 37°C without shaking. One-milliliter samples were extracted after 10, 20, 30, 45, 60, 75, and 90 min and treated as indicated above. Proteins were resolved by Tris-Tricine-PAGE (12%) electrophoresis and transferred onto polyvinylidene-difluoride membranes using a semidry electrophoresis transfer apparatus (Bio-Rad). Specific proteins were detected with anti-Flag M2 monoclonal antibody (1:5,000, Sigma-Aldrich) and anti-GroEL polyclonal antibody (1:20,000; Sigma-Aldrich). Goat anti-mouse horseradish peroxidase-conjugated antibody (1:5,000; Bio-Rad) or goat anti-rabbit horseradish peroxidase-conjugated antibody (1:20,000; Santa Cruz Biotechnology) were used as secondary antibodies. Proteins recognized by the antibodies were visualized by chemoluminiscence using the luciferin-luminol reagents (Thermo Scientific) in a LAS 3000 Mini Imaging System (Fujifilm). For quantification, the intensity of the hybridization bands was determined using MultiGauge software (Fujifilm). GroEL was used as loading control.

### RNA Extraction and Northern Blot Analysis

Aliquots from exponential cultures in LB and LB + 5% DOC were centrifuged at 16,000 × *g*, 37°C, during 2 min, and washed twice with 500 μl of NaCl 0.9%. The pellets were resuspended in 100 μl of a solution of lysozyme (Sigma-Aldrich), 3 mg ml^−1^. Cell lysis was facilitated by freezing at −20°C for >2 h. After lysis, RNA was extracted using 1 ml of TRIsure reagent (Bioline). Lastly, total RNA was resuspended in 25 μl of RNase-free water. The quality of the preparation and the RNA concentration were determined using a ND-1000 spectrophotometer (NanoDrop Technologies).

For Northern blot analysis, 10 μg of total RNA was loaded per well and electrophoresis was performed in denaturing 1% agarose-formaldehyde gels. Vacuum transfer and fixation to Hybond-N^+^ membranes (GE Healthcare) were performed using 0.05 M NaOH. UV crosslinking was used to immobilize RNAs on the membrane. For mRNA stability experiments, rifampicin (500 mg ml^−1^) was added to exponential cultures grown in LB at 37°C (zero-time control). At this point, the culture was divided into two types: the DOC sample, to which 25% of DOC was added to reach a final concentration of 5%, and the LB sample, to which LB was added to equalize the volume with the DOC sample. Incubation was continued, and culture aliquots were taken at appropriate times. DNA oligonucleotides (Racer2-zapB for *zapB* mRNA and 5S-probe for the loading control) were labeled with [^32^P]-γ-ATP using T4 polynucleotide kinase (Biolabs). Labeled probes were purified over microspin G-25 columns (GE Healthcare) to remove unincorporated nucleotides. As a control of RNA loading and transfer efficiency, the filters were hybridized with an oligoprobe for the 5S rRNA. Membranes were hybridized with oligoprobes at 42°C. Signals were visualized with a FLA-5100 image system (Fujifilm), and quantification was performed using MultiGauge software (Fujifilm).

### 5' Rapid Amplification of cDNA Ends

RNA was isolated from *S. enterica* stationary cells (DO_600_ ~2) grown in LB. Fifteen microgram of RNA was used to determine cDNA ends using a standard protocol. RNAs were prepared either with or without tobacco acid pyrophosphatase (TAP) to distinguish primary transcript 5' ends from internal 5' processing sites. The sequences of the primers are listed in Supplementary Table S1. The DNA primer for cDNA synthesis, Racer2-zapB, is complementary to *zapB* RNA. A second DNA primer for subsequent PCR amplification of cDNAs, Racer-out, is homologous to the adaptor RNA primer Racer-RNA-adaptor used for 5' rapid amplification of cDNA ends (5'-RACE). PCR products detected both with and without TAP treatment were further amplified with the internal primers Racer-nested and Racer1-zapB, and purified. PCR products were cloned on pGEM-T Easy (Promega), and 29 clones from two independent assays were sequenced.

### Site-Directed Mutagenesis of *zapB* and *micA*

Introduction of point mutations into MicA sRNA was performed by PCR using as template the wild type *micA* gene cloned on pBR328. Two pairs of primers were employed: MicA5101319-FOR and micAREVSalI; and MicA5101319-REV and micAFORBamHI. MicA5101319-FOR and MicA5101319-REV are complementary primers that introduce the point mutations in the gene, while micAREVSalI and micAFORBamHI introduce *ad hoc* restriction sites. The resulting DNA fragments were ligated into one using Gibson assembly (New England Biolabs, Ipswich, MA, United States). The fragment was digested with SalI and BamHI and cloned onto pBR328. The plasmid containing substitutions in MicA sRNA (5, G→C; 8, G→C; 13, U→A; and 19, A→U) was transformed into the *zapB* + 18::*lacZ* background to yield strain SV9486 (*zapB* + 18::lacZ pBR328-MicA*).

To insert point mutations into the chromosomal *zapB* gene, PCR was performed as described for MicA, using the pairs of primers For-zapB(MicA)-50,545,661 and zapB-XbaI-REV; and Rev-zapB(MicA)-50,545,661 and zapB-SacI-FOR. As above, ligation by Gibson assembly was followed by digestion and cloning onto pDMS197. The strains carrying pMDS197 derivatives were used as donors in matings with the *S. enterica* strain SV9265 (which harbors a Km^R^ cassette replacing the *zapB* coding sequence) as recipient. Tc^R^ transconjugants were selected on M9 plates supplemented with tetracycline. Several Tc^R^ transconjugants were grown in nutrient broth containing 5% sucrose. Individual tetracycline-sensitive segregants were then screened for kanamycin sensitivity and examined for the incorporation of the mutant *zapB* allele by DNA sequencing using external oligonucleotides (zapB-E1 and zapB-E2). The resulting mutant (SV9647, zapB*50A54T58A61G) contained four substitutions (50: U→A, 54: A→U, 58: U→A, and 61, C→G) that restored complementation with mutant MicA.

### *In vitro* Transcription and Gel Mobility Shift Assay

DNA templates for *in vitro* transcription of *zapB* mRNA and MicA sRNA were generated by PCR using chromosomal DNA from both the wild type and a *zapB* mutant (SV9647), and plasmid DNA from strains containing wild type and mutant variants of sRNA MicA cloned onto pBR328. The primers used were Transc-zapB-FOR and Transc-zapB-REV, and Transc-micA-FOR and Transc-micA-REV (Supplementary Table S1). The PCR products were purified with the Wizard® SV Clean-Up System (Promega). For the *in vitro* transcription reaction, the HiScribe T7 High Yield RNA Synthesis kit was employed. Two sets of reactions were prepared: (i) for synthesis of non-labeled *zapB* transcripts, the *zapB* PCR product was mixed with equimolar concentrations of all four ribonucleotides (10 mM) and (ii) for synthesis of internally labeled MicA sRNA and *zapB* mRNA, each PCR product was mixed with ATP, GTP, and CTP 10 mM and 0.4 mM of UTP. Radioactive labelling was performed by adding 3 μl of EasyTide® uridine 5'-triphosphate (α-^32^P) 3,000 Ci mmol^−1^ (PerkinElmer) to a final concentration of 0.25 μM. One microliter of T7 RNA Polymerase Mix and 4 μl of Master Mix were added to each reaction tube. Nuclease-free water was added to a final volume of 40 μl. The reactions were then incubated at 37°C for 2 h and treated with DNase (TURBO DNase Ambion 2 U μl^−1^) for 15–20 min. To discard unincorporated nucleotides, the reactions were passed through GE Healthcare ilustra™ MicroSpin™ G-25 columns. Subsequently, the RNA transcripts (both labeled and unlabelled) were purified by electrophoresis on a 7.9 M urea, 8% polyacrylamide denaturing gel. The hybridization fragments were visualized using flour-coated TLC plates (Ambion). Gel slices were crushed, and RNA was eluted overnight at 4°C with crush-soak solution (200 mM NaCl, 10 mM Tris-HCl, and 10 mM EDTA). The RNA was ethanol-precipitated and resuspended in nuclease-free water.

Gel mobility shift assays were performed with 0.015 pmol of [α-^32^P]-labeled wild type or mutant MicA (MicA*) RNA, and 0.075 pmol of unlabelled *zapB* mRNA transcript in 1× binding buffer (200 mM Tris-HCl pH 8, 10 mM DTT, 10 mM MgCl_2_, 200 mM KCl, and 100 mM Na_2_HPO_4_-NaH_2_PO_4_) at 37°C. The reactions were incubated, and samples were taken at 10, 30, 60, and 100 min. The binding reactions were mixed with 2 μl of loading dye (48% glycerol and 0.01% bromophenol blue) and subjected to electrophoresis at room temperature in a 6% non-denaturing polyacrylamide Tris-borate EDTA (TBE) gel for 2 h at 55 V, 15 mA in 1x TBE buffer (90 mM Tris-borate/2 mM EDTA). In parallel to the binding reactions, a positive control was prepared by mixing *zapB* mRNA and [α-^32^P]-labeled MicA (mutant and wild type) followed by boiling denaturation and renaturation at 37°C. Single [α-^32^P]-labeled *zapB* RNA and MicA were also loaded on the gel. After electrophoresis, gels were dried and analyzed in a FLA-5100 Scanner (Fujifilm).

### Statistical Analysis

Student’s *t*-test (unpaired) was performed using GraphPad Prism version 8.0 for Mac to determine the statistical differences between two groups. Ordinary one-way ANOVA was also performed using GraphPad Prism.

## Results

### The ZapB and ZapA Proteins of *S. enterica* Are Necessary for Bile Resistance

The *zapB* gene of *E. coli* encodes an 81-amino-acid protein that acts as a non-essential cell division factor involved in Z-ring assembly (Adams and Errington, 2009). The name *zapB* is for “Z ring-associated protein B” (Ebersbach et al., 2008). Alignment of the amino acid sequences of the ZapB protein of *E. coli* and the predicted protein encoded by the *yiiU* locus of *Salmonella enterica* reveals a 91% identity value. For this reason, the *S. enterica yiiU* locus will be henceforth named *zapB*.

Minimal inhibitory concentration (MIC) analysis was performed to determine whether the *S. enterica* ZapB protein is necessary for bile resistance. A null ZapB^−^ mutant (SV6269) was found to be sensitive to DOC (Table 2). The conclusion that ZapB is necessary for bile resistance was confirmed by complementation analysis with plasmid-borne *E. coli* and *S. enterica zapB* genes expressed from the arabinose-inducible promoter of pBAD18 (Table 2).

**Table 2:** Minimal concentrations of sodium deoxycholate (DOC)^a^.

Strain	Genotype	MIC of DOC (%)
SL1344	Wild type	7
SV6898	*zapB*::3 × FLAG	7
SV6269	*∆zapB*	2
SV7264	SL1344/pBAD18	7
SV7265	*∆zapB*/pBAD18	2
SV7267	SL1344/pBAD18::*zapB*	7
SV7268	*∆zapB*/pBAD18::*zapB*	7
SV7432	*∆zapB*/pBAD18::*zapB E. coli*	7
SV7016	*Lon* Tn*10*	9
SV7022	*zapB::*Km^R^ *lon::*Tn*10*	2
SV7300	*zapA*::Km^R^	4
SV9770	*ΔzapA ΔzapB*	2
SV9771	SL1344/pBAD18::*zapA*	7
SV9772	*ΔzapA*/pBAD18	4
SV9773	*ΔzapA*/pBAD18::*zapA*	7
SV9775	*ΔzapA*/pBAD18::*zapA E. coli*	7

a Medians of 4–6 experiments.

The observation that a ZapB^−^ mutant is sensitive to bile raised the possibility that lack of another Z-ring assembly modulator, ZapA, might also cause bile sensitivity. A bioinformatic search followed by Clustal W alignment revealed that the *ygfE* gene of *S. enterica* encodes a protein with 88% amino acid identity with *E. coli* ZapA. YgfE was thus renamed ZapA. Disruption of the *zapA* gene caused sensitivity to DOC, and complementation tests using plasmid-borne *E. coli* and *S. enterica zapA* genes restored bile resistance (Table 2).

The MIC of DOC for a *zapA zapB* mutant was similar or identical to that of a *zapB* mutant, an epistatic effect which suggests that bile sensitivity in the absence of either ZapA or ZapB results from disruption of the same cellular process.

### Expression of *zapB* Is Upregulated in the Presence of Bile

The levels of *zapA* and *zapB* mRNAs in LB and LB + DOC were monitored by qPCR. Relative RNA levels, averaged from six independent experiments, were as follows: *zapA*, 1.00 (arbitrary) in LB and 1.21 ± 0.36 in LB + DOC; and *zapB*, 2.09 ± 0.49 in LB and 8.80 ± 1.78 in LB + DOC. These experiments confirmed previous observations indicating that expression of *zapB* (but not of *zapA*) increases in the presence of bile (Hernandez et al., 2012). Further work was centered on *zapB* only.

### The ZapB Protein Is Degraded by the Lon Protease in the Presence of Bile

**Figure 1:**
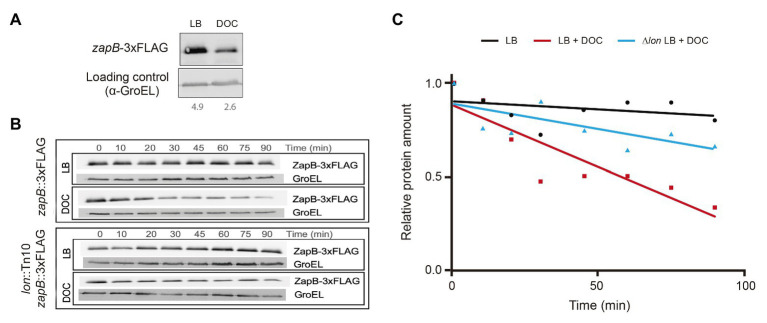
Degradation of ZapB protein by the Lon protease in the presence of DOC. **(A)** Western blot analysis of the levels of ZapB-3 × FLAG protein in lysogeny broth (LB) and LB + 5% DOC. **(B)** Stability of the ZapB-3 × FLAG protein in exponential cultures grown in LB and LB + 5% DOC in wild type and Lon^−^ backgrounds. Aliquots were extracted 10, 20, 30, 45, 60, 75, and 90 min after addition of DOC and chloramphenicol. **(C)** Graphical representation of the data of panel B, showing the stability of the ZapB-3 × FLAG protein in LB and LB + DOC and the effect of a *lon* mutation.

The stability of the ZapB protein in the presence and in the absence of DOC was analyzed by Western blot analysis using a ZapB version tagged with the 3 × FLAG epitope (ZapB::3 × FLAG). Note that the strain carrying this ZapB variant is resistant to DOC and the protein may be, therefore, considered wild type (Table 2). The amount of ZapB protein detected in LB + DOC was lower than in LB (Figure 1A), suggesting that ZapB might be degraded in the presence of DOC. To investigate this possibility, we carried out protein stability assays to monitor the half-life of the ZapB::3 × FLAG protein upon addition of DOC. As shown in Figures 1B,C, ZapB is degraded faster in LB + DOC (half-life around 30 min) than in LB (half-life >90 min).

Because the Lon protease is involved in the degradation of unfolded and misfolded proteins (Tsilibaris et al., 2006; Gur et al., 2011) and bile salts cause protein misfolding and denaturation (Bernstein et al., 1999; Leverrier et al., 2003), the stability of ZapB protein was monitored in a Lon^−^ background (strain SV7017). In the absence of Lon protease, the ZapB stability in LB + DOC increased (Figures 1B,C). Hence, we tentatively conclude that the Lon protease may degrade ZapB in the presence of DOC.

### Postranscriptional Regulation of *zapB*

**Figure 2:**
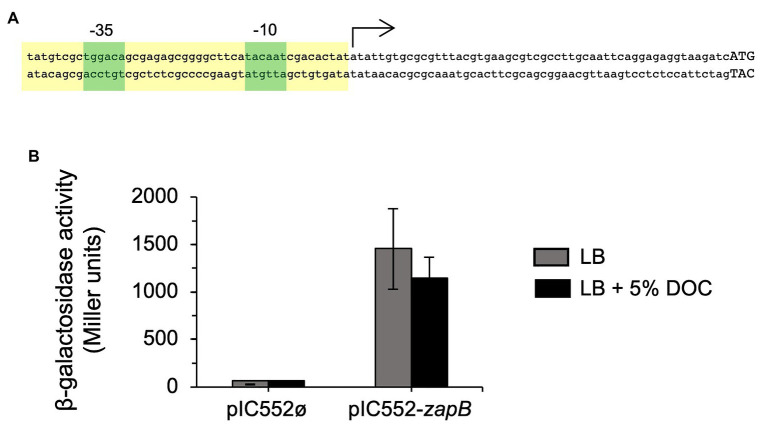
Identification of the *zapB* promoter. **(A)** Diagram of the putative promoter region of *zapB*. The transcription initiation site (arrow), and the putative −35 and −10 modules (green boxes) are shown. Capital letters are nucleotides of the putative *zapB* coding sequence. The region that was cloned on the promoter-probe vector pIC552 to generate a transcriptional fusion with the *lacZ* gene is outlined in yellow. **(B)** β-Galactosidase activities of strain SV7339 (carrying the pIC552-*zapB* derivative) in LB and in LB + 5% sodium DOC. Strain SV7338 (carrying the empty vector) was included as control. Data are averages and SD from three independent experiments.

To investigate the mechanism underlying *zapB* upregulation in the presence of DOC, the transcriptional start site of the *zapB* gene was identified by 5'-RACE. Twenty-four out of twenty-nine clones analyzed from two independent 5'-RACE assays identified a 5'-end A residue located 56 nucleotides upstream of the *zapB* translational start codon. *In silico* analysis of the region revealed the occurrence of nucleotide sequences compatible with the consensus sequences of −35 and −10 modules in *σ*^70^-dependent promoters, and with appropriate spacing (Collado-Vides, 1996). The proposed structure of the *zapB* promoter is presented in Figure 2A.

To confirm the existence of the predicted *zapB* promoter, the putative promoter region was cloned on the promoter-probe vector pIC552 (Macian et al., 1994) to generate a transcriptional fusion with the *lacZ* gene. The pIC552 derivative bearing the *zapB* promoter was introduced into the wild type strain, and β-galactosidase activity measurements confirmed that the cloned region was able to drive *lacZ* expression. Interestingly, transcription of the pIC552-borne p*_zapB_*-*lacZ* gene was not regulated by DOC (Figure 2B). This observation raised the possibility that upregulation of *zapB* expression in the presence of DOC might be postranscriptional.

**Figure 3:**
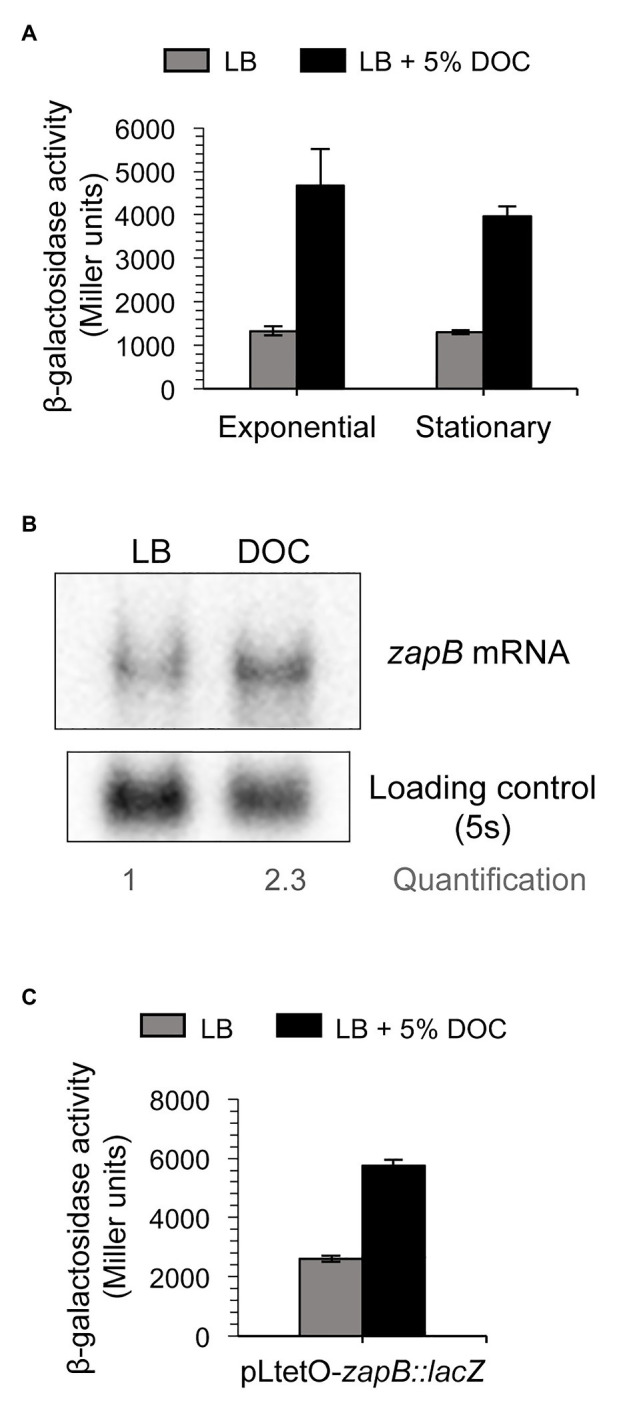
Evidence for postranscriptional control of *zapB*. Regulation of *zapB* expression by bile. **(A)** β-Galactosidase activity of the translational *zapB::lacZ* fusion of strain SV6270 in exponential and stationary cultures in LB (gray histograms) and LB + 5% DOC (black histograms). **(B)** Northern blot analysis of the levels of *zapB* mRNA in extracts from stationary cultures in LB and LB + 5% DOC. **(C)** β-Galactosidase activity of a *zapB::lacZ* fusion under the control of the p_LtetO_ promoter (strain SV7429) in LB (gray histograms) and LB + 5% DOC (black histograms).

Upregulation of *zapB* mRNA in the presence of bile was confirmed by β-galactosidase assays using a translational *zapB::lacZ* fusion and by Northern blot analysis (Figures 3A,B). When the translational *zapB::lacZ* fusion was placed under the control of a heterologous promoter, pL_tetO_ (Lutz and Bujard, 1997), increased *zapB* expression was still observed in the presence of DOC (Figure 3C). This observation was in agreement with the above observation that the *zapB* promoter cloned on pIC552 was not responsive to DOC (Figure 2B), leading us to conclude that DOC-dependent regulation of *zapB* is not transcriptional.

### Bile Stabilizes *zapB* mRNA by a Mechanism Involving Hfq

**Figure 4:**
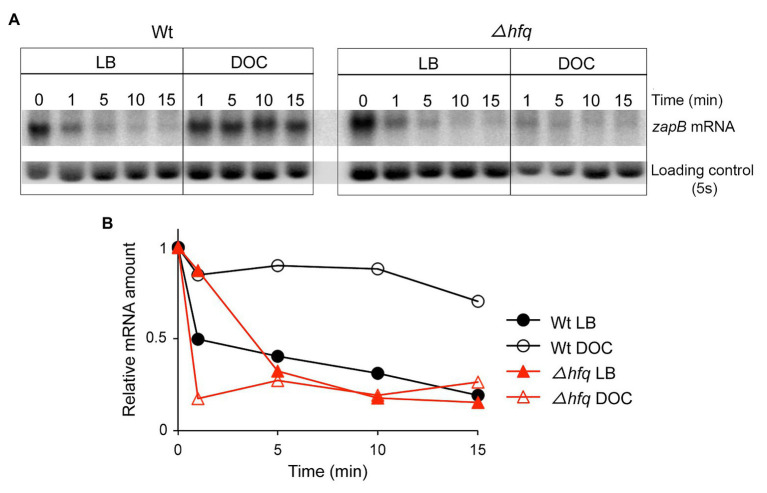
Stability of *zapB* mRNA. **(A)** Levels of *zapB* mRNA in RNA extracts from exponential cultures in LB and LB + 5% DOC. Samples were taken 1, 5, 10, and 15 min after addition of rifampicin. **(B)** Quantification of the level of *zapB* mRNA relative to the loading control and to time zero.

Evidence for postranscriptional upregulation of *zapB* in the presence of bile prompted a comparison of the stability of the *zapB* transcript in LB and in LB + DOC. For this purpose, Northern blot analysis was performed. Decay of *zapB* mRNA was faster in LB than in LB + DOC (Figure 4), suggesting that the higher amount of *zapB* mRNA found in the presence of DOC might result from increased mRNA stability.

Among the mechanisms that control bacterial mRNA stability, interaction with small regulatory RNAs (sRNAs) is common (Storz et al., 2004, 2011; Caron et al., 2010; Podkaminski and Vogel, 2010; Prevost et al., 2011). Hence, we considered the possibility that the stability of *zapB* mRNA might be controlled by a sRNA, either destabilizing the transcript in LB or stabilizing the transcript in the presence of DOC. Because most bacterial small regulatory RNAs require the Hfq chaperone for interaction with the target and for stability of the sRNA itself (Valentin-Hansen et al., 2004; McCullen et al., 2010; Soper et al., 2011), we analyzed the stability of *zapB* mRNA in an Hfq^−^ strain using Northern blotting (Figure 4). In the Hfq^−^ background, the stability of the *zapB* transcript with and without DOC was similar to the stability of *zapB* mRNA in the wild type strain grown in LB, suggesting that stabilization of *zapB* mRNA in the presence of DOC requires the Hfq RNA chaperone (Figures 4A,B).

### Identification of the *zapB* mRNA Target Region Involved in Postranscriptional Control

**Figure 5:**
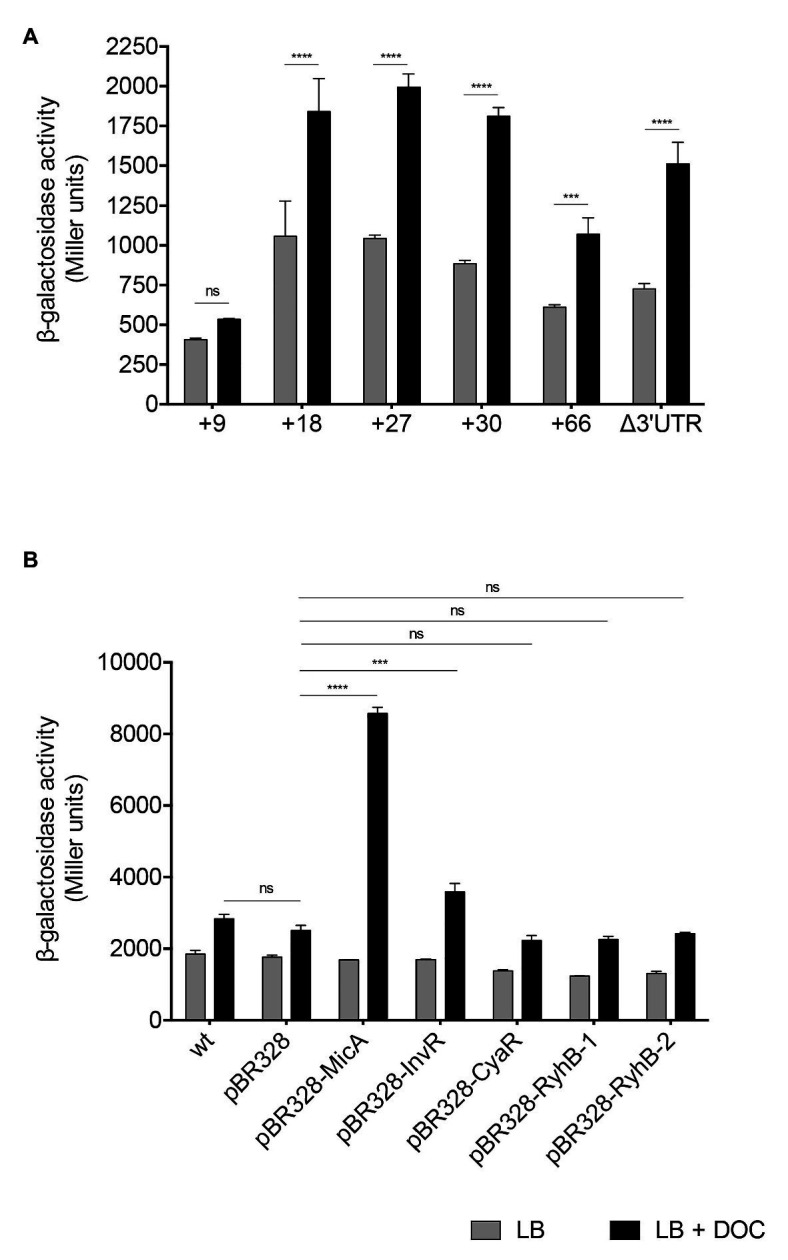
Identification of a *zapB* region involved in regulation by DOC. **(A)** β-Galactosidase activities of *zapB::lacZ* fusions inserted 9, 18, 27, 30, and 66 nucleotides downstream of the start codon of the *zapB* coding sequence, and of a *zapB::lacZ* fusion lacking the 3'UTR of the *zapB* transcript. **(B)** β-Galactosidase activities of *zapB::lacZ* fusions in strains overexpressing the sRNAs MicA, InvR, CyaR, RyhB-1, and RyhB-2 cloned onto the pBR328 plasmid. As controls, the *zapB::lacZ* strain (SV6270, named wild type) and a strain carrying the empty vector were assayed. The strains were grown in LB (gray histograms) and in LB + 5% DOC (black histograms). Statistical indications: ns, not significantly different; significantly different, ****p < 0.001; ***p < 0.01.

To identify the hypothetical *zapB* mRNA region that might be subjected to postranscriptional control, we constructed a set of translational fusions inserting the *lacZ* gene at different locations within the *zapB* coding sequence: 9, 18, 27, 30, and 66 nucleotides after the start codon (Supplementary Figure S1). Because 3'UTRs are often targets for sRNA binding (Opdyke et al., 2004; Papenfort and Vanderpool, 2015), we also constructed a strain in which the 3'UTR of *zapB* was deleted downstream of the *zapB::lacZ* fusion (Supplementary Figure S1). Measurement of β-galactosidase activities upon growth in LB and in LB + DOC provided the following observations:

1. The *zapB::lacZ* fusion that conserves only nine nucleotides of the *zapB* coding region showed reduced expression in both LB and LB + DOC (Figure 5A) and was not regulated by DOC.
2. Deoxycholate-dependent regulation was observed in the remaining *lacZ* fusions (Figure 5A), including the strain that maintains 18 nucleotides of the *zapB* coding region, suggesting that the upstream (5') 18 nucleotides of the *zapB* mRNA coding region are required for regulation by DOC.
3. The 3'UTR was found to be dispensable for *zapB* regulation by DOC (Figure 5A).

Altogether, the above observations provide evidence that the 5'UTR and the upstream (5') region of the *zapB* coding sequence (CDS) may be the main (perhaps the only) region of *zapB* mRNA involved in postranscriptional control by DOC.

### Search for Small Regulatory RNA(s) That Interact With *zapB* mRNA

We did not rule out the possibility that Hfq binding might be sufficient to stabilize *zapB* mRNA. However, this possibility seemed unlikely as Hfq often acts in concert with sRNAs (Basineni et al., 2009; Chao and Vogel, 2010; Soper et al., 2010; Storz et al., 2011). A computational search for potential interactions between *Salmonella* sRNA candidates and *zapB* mRNA using the IntaRNA program (Smith et al., 2010) identified sRNAs with potential targets in *zapB* mRNA (Supplementary Table S2). Such sRNA candidates were shortlisted using two criteria: (i) the potentially interacting mRNA sequence should be located in the 5' region of the *zapB* transcript and (ii) the potentially interacting sRNA should be regulated by bile. These criteria were met by five candidates: MicA, InvR, CyaR, RyhB-1, and RyhB-2, previously reported to be upregulated in the presence of bile (Kroger et al., 2013).

MicA, InvR, CyaR, RyhB-1, and RyhB-2 sRNAs were cloned onto pBR328, and the resulting plasmids were transformed into a *Salmonella* strain harboring a *zapB::lacZ* fusion. β-Galactosidase activities in LB and in LB + DOC were then determined (Figure 5B). The presence of DOC resulted in higher β-galactosidase activities when the sRNAs MicA and InvR were overexpressed, and the MicA effect was especially strong. The other sRNAs tested in the assay did not show any effect. These results suggest that MicA may be involved in stabilization of *zapB* mRNA in the presence of DOC.

### Interaction Between *zapB* mRNA and MicA

**Figure 6:**
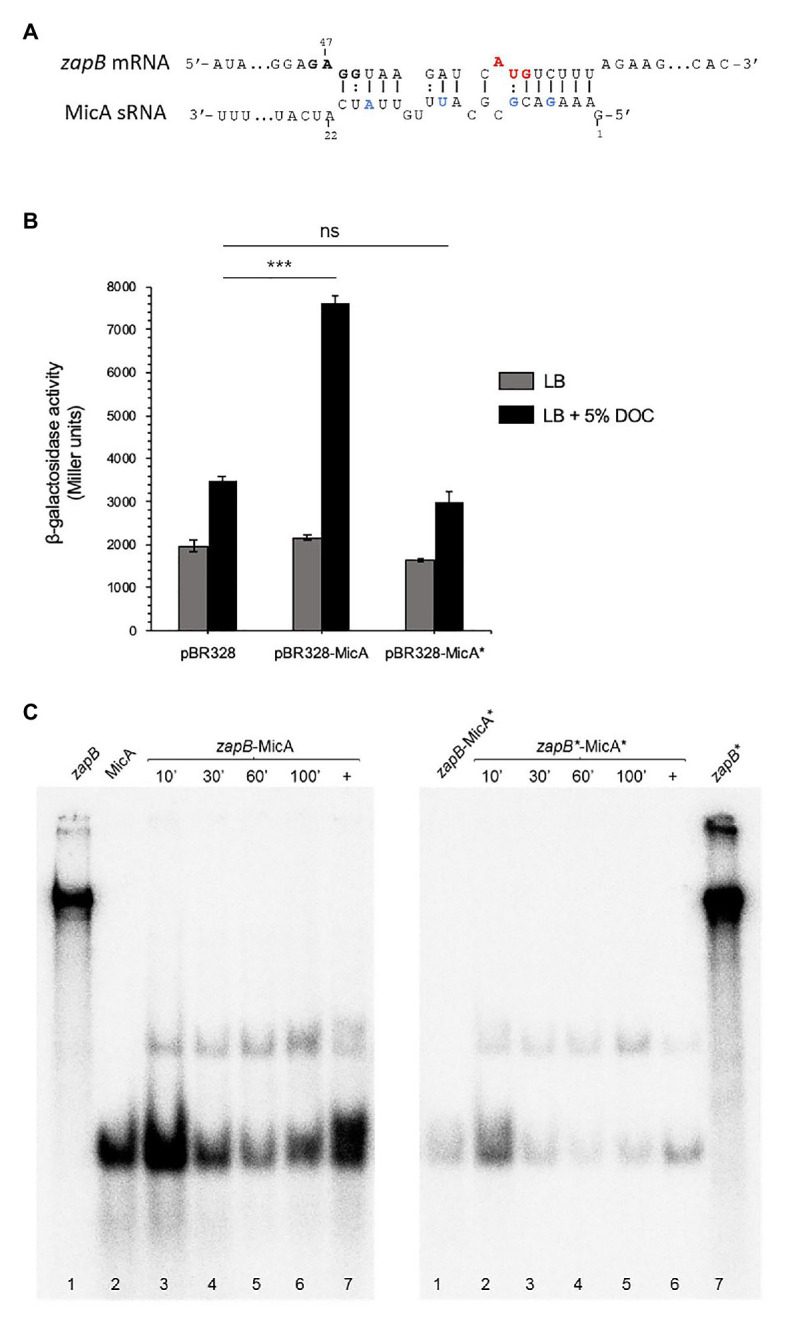
*zapB*-MicA interaction. **(A)** Diagram of putative interacting regions in sRNA MicA and *zapB* mRNA predicted by IntaRNA. The putative ribosome binding site and start codon are outlined (bold and red, respectively). MicA nucleotides subjected to site-specific mutagenesis are outlined in blue. **(B)** β-Galactosidase activities of *zapB::lacZ* fusions in strains overexpressing either MicA or MicA*. As controls, a *zapB::lacZ* strain (SV9372, “wild type”) and a strain carrying the empty vector were included. The strains were grown in LB (gray histograms) and in LB + 5% DOC (black histograms). **(C)** Gel mobility shift assay (EMSA) showing *in vitro* interactions between *zapB*-MicA and zapB*-MicA*. Samples were taken at 10, 30, 60, and 100 min. Lanes 1 and 2 in the left panel and lane 7 in the right panel show labeled *zapB*, MicA, and zapB* individual samples, respectively. As positive controls (+), *zapB* and MicA RNAs were treated to force association (lane 7, left and lane 6, right). As negative control, a mix of wild type *zapB* mRNA with mutant MicA (zapB-MicA*) was included (lane 1, right). Statistical indications: ns, not significantly different; significantly different, ***p < 0.01.

*In silico* analysis with the IntaRNA tool (Smith et al., 2010) predicts that MicA might interact with *zapB* mRNA by base pairing at a *zapB* mRNA region that contains a putative ribosome binding site (5'GAGG3') and the AUG start codon of the coding sequence (Figure 6A). To probe the predicted interaction, point mutations were introduced in MicA sRNA by site-directed mutagenesis: 5, G→C; 8, G→C; 13, U→A; and 19, A→U. The mutant version of *micA* (micA*) was then cloned on pBR328 and transformed into a strain carrying a *zapB::lacZ* fusion. Expression of *zapB::lacZ* in LB and in LB + DOC was compared with that of a strain that overexpressed wild type MicA. β-Galactosidase activity analysis indicated that the upregulation of the *zapB* transcript detected upon MicA overexpression disappeared when MicA was mutated (Figure 6B). Actually, the β-galactosidase activity in the presence of MicA* was similar to that of the control harboring the empty vector (Figure 6B). These observations suggest that the mutations introduced into MicA had disrupted the interaction with *zapB* mRNA, thus supporting the existence of a *zapB* mRNA:MicA sRNA interaction at the region predicted by bioinformatic analysis.

Further evidence for *zapB* mRNA:MicA sRNA interaction was obtained *in vitro*. An electrophoretic mobility shift assay (EMSA) was carried out using wild type and mutant versions of both *zapB* mRNA and MicA sRNA, the latter labeled with [α-^32^P] (Figure 6C). The mutations introduced in zapB*, the mutant version of *zapB* mRNA (50: U→A, 54: A→U, 58: U→A, and 61, C→G) were complementary to those present in MicA*, so that the interaction predicted by IntaRNA could be restored. Binding was detected for both *zapB* mRNA:MicA and zapB* mRNA:MicA*, and the fact that binding was largely independent from the incubation time may suggest a strong interaction (Figure 6C). Altogether, the experiments shown in Figures 5, 6 support the following tentative conclusions: (i) *zapB* mRNA and MicA sRNA do interact; (ii) the main (perhaps only) interaction involves the upstream region of *zapB* mRNA; and (iii) the *zapB*:MicA interaction increases *zapB* expression in the presence of DOC, probably by MicA-mediated stabilization of *zapB* mRNA.

## Discussion

Initial evidence that the Z-ring might be involved in bile resistance was provided by transcriptomic analysis: the *zapB* gene, which in *Salmonella* was still annotated as a locus of unknown function (*yiiU*), was found to be upregulated in the presence of a sublethal concentration of DOC (Hernandez et al., 2012). Disruption of the *yiiU* locus causes sensitivity to DOC (Table 1), indicating that *yiiU* is not merely a bile-induced locus but a gene necessary for bile resistance. Lack of the *S. enterica ygfE* gene product likewise causes bile sensitivity (Table 1). Change of the *ygfE* and *yiiU* gene designations to *zapA* and *zapB* is supported by the 88 and 91% identity between the predicted *Salmonella* and *E. coli* gene products, respectively. Unlike *zapB*, the *S. enterica zapA* gene is not upregulated by bile.

Upregulation of *S. enterica zapB* expression in the presence of DOC is still observed when *zapB* transcription is driven by a heterologous promoter (Figure 3C), thereby suggesting the involvement of a postranscriptional mechanism. Comparison of *zapB* mRNA decay in LB and LB + 5% DOC reveals that *zapB* mRNA is more stable in the presence of DOC, thus explaining the higher mRNA level detected both in the initial transcriptomic analysis (Hernandez et al., 2012) and in Northern blots (Figures 3B, 4). Stabilization of the *zapB* transcript in the presence of DOC requires the Hfq RNA chaperone (Figure 4), an effect that admits two alternative explanations: (i) Hfq binding might protect *zapB* mRNA from degradation and (ii) Hfq might catalyze the interaction of *zapB* mRNA with a small regulatory RNA, and the mRNA:sRNA interaction might increase *zapB* mRNA stability.

Construction of *lacZ* fusions in truncated forms of the *zapB* transcript defined the 5'UTR and the upstream (5') 18 nucleotides of the coding sequence as a region necessary for upregulation in the presence of bile salts (Figure 5A). This interpretation is in agreement with the fact that activating sRNAs often target upstream mRNA regions (Papenfort and Vanderpool, 2015).

*In silico* search for sRNAs that might interact with *zapB* mRNA by base pairing provided the candidates listed in Supplementary Table S2. Upregulation in the presence of bile (Kroger et al., 2013) and putative interaction at the 5' region of *zapB* narrowed the number of candidate sRNAs down to five: CyaR, InvR, MicA, RyhB-1, and RyhB-2. When overexpression of these sRNAs was tested, MicA conspicuously increased the β-galactosidase activity of a *zapB::lacZ* fusion in the presence of DOC (Figure 5B). Occurrence of a *zapB* mRNA:MicA sRNA interaction was further supported by the existence of a potential base pairing region (Figure 6A) and by the fact that introduction of point mutations in MicA abolished *zapB::lacZ* upregulation in the presence of DOC (Figure 6B). Interaction between *zapB* mRNA and MicA sRNA was also detected *in vitro* (Figure 6C). Furthermore, site-directed mutagenesis of MicA disrupted the interaction, which was restored upon introduction of compensatory mutations in *zapB* mRNA (Figure 6C).

MicA is a small regulatory RNA that participates in the bacterial envelope stress response mediated by the sigma factor RpoE (Figueroa-Bossi et al., 2006). MicA binds *omp* mRNAs accelerating their decay and enabling outer membrane remodeling upon envelope stress (Papenfort et al., 2006; Bossi and Figueroa-Bossi, 2007). Identification of additional mRNA targets has linked MicA to the *phoPQ* regulon and to additional functions (Coornaert et al., 2010; Kint et al., 2010; Gogol et al., 2011; Van Puyvelde et al., 2015). Our observation that MicA protects the *zapB* transcript from degradation in the presence of DOC departs from previous examples of MicA-mediated regulation, which involved stimulation of mRNA turnover (Papenfort et al., 2006; Bossi and Figueroa-Bossi, 2007). This difference is further emphasized by the observation that the region of MicA involved in protection of *zapB* mRNA (nt 2–21) overlaps with regions involved in MicA-mediated turnover in other mRNAs: 1–22 in *ompX* (Gogol et al., 2011) and 8–24 in *ompA* (Udekwu et al., 2005). These mechanistic differences may seem paradoxical. However, MicA-mediated stabilization of *zapB* mRNA in the presence of bile is anything but paradoxical: a known role of MicA in the bacterial cell is protection from envelope stress (Figueroa-Bossi et al., 2006; Papenfort et al., 2006; Bossi and Figueroa-Bossi, 2007). On the other hand, transcript stabilization upon interaction with the mRNA 5' region has been described for other sRNAs (Papenfort and Vogel, 2009; Storz et al., 2011).

Evidence that ZapB is degraded by the Lon protease in the presence of DOC is presented in Figure 1. Lon degrades abnormally folded proteins (Gottesman, 1996), and bile salts cause misfolding and denaturation of proteins (Bernstein et al., 1999). We thus propose that bile salts may cause ZapB misfolding, and that misfolding may trigger degradation by Lon. If this view is correct, increased *zapB* mRNA stability in the presence of bile may compensate for Lon-mediated degradation. Stabilization of *zapB* mRNA may be in turn facilitated by the existence of a higher concentration of MicA in the presence of bile (Kroger et al., 2013).

The epistatic effect of a *zapB* mutation over a *zapA* mutation suggests that the ZapA and ZapB gene products participate in the same cellular process. Because ZapA and ZapB are Z-ring assembly modulators (Adams and Errington, 2009), we tentatively propose that perturbation of Z-ring assembly may render the *Salmonella* cell bile-sensitive. The immediate cause of bile sensitivity in the absence of Z-ring modulators remains unknown. However, it is remarkable that another cell division factor, DamX, is also necessary for bile resistance in both *S. enterica* (Lopez-Garrido et al., 2010) and *E. coli* (Arends et al., 2010).

## Data Availability Statement

The raw data supporting the conclusions of this article will be made available by the authors, without undue reservation.

## Author Contributions

RF-F, SH, VU, and MS-R carried out the experiments. RF-F, SH, EP-F, MS-R, VU, and JC designed the experiments and interpreted results. JC wrote the manuscript. All authors contributed to the article and approved the submitted version.

### Conflict of Interest

The authors declare that the research was conducted in the absence of any commercial or financial relationships that could be construed as a potential conflict of interest.

## Footnotes

^1^http://www.universalprobelibrary.com
