# Supplementary material for: Evidence for Involvement of the Salmonella enterica Z-Ring Assembly Factors ZapA and ZapB in Resistance to Bile
Source: Front Microbiol. 2021 Feb 25;12:647305. doi: 10.3389/fmicb.2021.647305 (PMC7947894; doi:10.3389/fmicb.2021.647305)
Supplement: Supplementary file 1 [file Data_Sheet_1.pdf]

Evidence for involvement of the *Salmonella enterica* Z-ring assembly factors ZapA and ZapB in resistance to bile

Rocío Fernández-Fernández, Sara B. Hernández, Elena Puerta-Fernández,

María A. Sánchez-Romero, Verónica Urdaneta, and Josep Casadesús

SUPPLEMENTARY MATERIAL

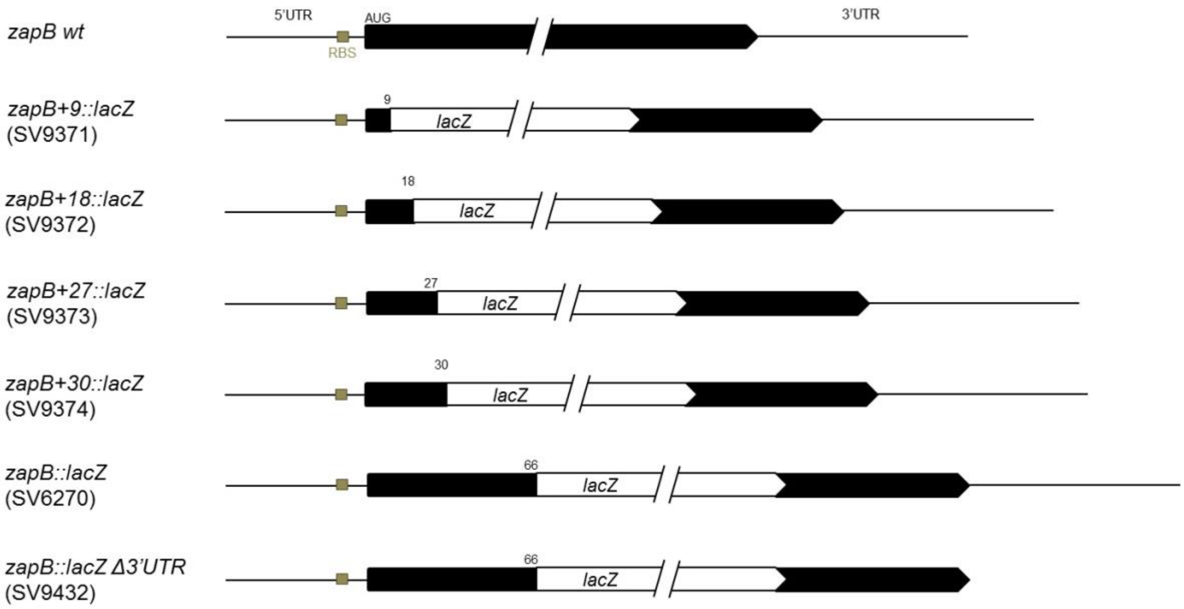

Figure S1. Diagrams of wild type *zapB* mRNA and of *lacZ* translational fusions at different locations within the *zapB* coding sequence. The 5'UTR is 56 nt long, and includes a putative ribosome binding site (GAGG, +46 to +49) in the region of interaction with MicA. The coding sequence is 240 nt long, and the predicted length of the 3'UTR (not drawn at scale) is 113 nt.

Table S1. Oligonucleotides used in this study (5´-3')

pIC-zapB-FOR	TACGAGATCTGCAAACCGCGATCTACGCGGTATGTC
pIC-zapB-REV	TACGCTCGAGGAATTGCAAGGCGACGCTTCACGTAAAC
Racer1-zapB	GGGTGATGGTGTCAATCGCCTGC
Racer2-zapB	CAATCGCCTGCTGTACTTTTGCTTCC
Racer-nested	GGACACTGACATGGACTGAAGGAGTA
Racer-Out	CGACTGGAGCACGAGGACACTGA
Racer-RNA-adaptor	CGACUGGAGCACGAGGACACUGACAUGGACUGAAGGAGUAGAAA
zapB::mChe-pBAD-REV	TCGAGTCGACGCAGCCAACGCCGCCATAACGTGAAATATGACGAGGATTAAGTCACGACG
zapB-E1	TGAGACAGCAGAAGAGAGTG
zapB-E2	CCGCTTGATATCGAGTGATC
zapBEcoli-pBAD-FOR	ATCGGAATTCCAATTCAGGAGAGGTATGACAATGTC
zapBEcoli-pBAD-REV	TCGAGTCGACCGGAAGATGAAGCGTAATCAGACCTC
zapB-FLAG-FOR	GGAACGTTTGCAGGCGCTGCTTGGTCGTATGGAAGAAGTCGACTACAAAGACCATGACGG
zapB-FLAG-REV	CACGCAGCCAACGCCGCCATAACGTGAAATATGACGAGGACATATGAATATCCTCCTTAG
zapB-MChe-FOR	GGAACGTTTGCAGGCGCTGCTTGGTCGTATGGAAGAAGTCATGGTGAGCAAGGGCGAGGA
zapB-MChe-REV	GCAGCCAACGCCGCCATAACGTGAAATATGACGAGGATTAAGTCACGACGTTGTAAAACG
yiiU-P1-REV	AAACGTTCCTGCCAGCCGCTCTGCTGTTCCTTCAGAGAATGTGTAGGCTGGAGCTGCTTC
yiiU-P4-FOR	GGAAGCAAAAGTACAGCAGGCGATTGACACCATCACCCTGATTCCGGGGATCCGTCGACC
zapB-CDS-P4-FOR	TACGTGAAGCGTCGCCTTGCAATTCAGGAGAGGTAAGATCATTCCGGGGATCCGTCGACC
zapB-CDS-P1-REV	GCCAACGCCGCCATAACGTGAAATATGACGAGGACA

	CGCAGTGTAGGCTGGAGCTGCTTC
zapB-P4-FOR(+9)	CGTCGCCTTGCAATTCAGGAGAGGTAAGATCATGTC TTTAATTCCGGGGATCCGTCGACC
zapB-P4-FOR(+18)	GCAATTCAGGAGAGGTAAGATCATGTCTTTAGAAGT GTTTATTCCGGGGATCCGTCGACC
zapB-P4-FOR(+27)	GAGAGGTAAGATCATGTCTTTAGAAGTGTTTGAGAAA CTGATTCCGGGGATCCGTCGACC
zapB-P4-FOR(+30)	AGGTAAGATCATGTCTTTAGAAGTGTTTGAGAAACTG GAAATTCCGGGGATCCGTCGACC
zapA-E1	AGCATTCGTCATCAAAGGGAGG
zapA-E2	AGGTGAATGTGTCGTCACAG
zapAEC-E1	GCAATGTCTGCACAACCCGTCGATATCCAAATTCGAT
zapAEC-E2	ACCGAAAAAACTAACCAAAACTTTGAAGTCCCAT
MICAFORBAMHI	ATCGGGATCCTGTTCGGAATGCAAAAACGCAGATCA AACA
MICAREVSALI	ATCGGTCGACATTGATGAAGGCATTGGCGGCACCG GGAAA
INVRFORBAMHI	ATCGGGATCCAGAGCAACTCATGACCGAATTTAAAA AAGT
INVRREVSALI	ATCGGTCGACTACTAGCCTGGTCGTCAATACGCTGT CACG
RYHB-1FORBAMHI	ATAGGGATCCACACCTCCGGCTGGGCGTGGATCTG ACGAA
RYHB-1REVSALI	ATCGGTCGACAGACTGAATGTTGGATTCTTTGCCGGAT[illegible]
CYARFORBAMHI	ATCGGGATCCACCTGGCGATGGCTATACCGTCTGGAT GCC
CYARREVSALI	AGCGCGTGACGTTATTGATTTAATTCACTGATTTTAAA CG
PBR328-E1	ACTGTCCGACCGCTTTGG
PBR328-E2	GCCAGCAACCGCACCTG
TRANSC-ZAPB-FOR	TAATACGAGTCACTATAGGATATATTGTGCGCGTTTAC GTGAAGC

TRANSC-ZAPB-REV	GTGAATCCCCAGGAGCATAGG
TRANSC-MICA-FOR	TAATACGACTCACTATAGGAAAGACGCGCATTTGTTATC
TRANSC-MICA-REV	AAAAAGGCCACTCACGGAG
3UTRZAPB-P2-REV	ATCCTCTATAGATTTCGCGTCAGGGCAAGGCGGCAAGTAACATATGAATAT
MICA5101319-FOR	GTCTGAGTATATGAAACACGCCCAATTGTTTTCATCATCCCTG
MICA5101319-REV	CAGGGATGATGAAAACAATTGGGCGTGTTTCATATACTCAGAC
MICAFORBAMHI	ATCGGGATCCTGTTCGGAATGCAAAAACGCAGATCAAACA
MICAREVSALI	ATCGGTCGACATTGATGAAGGCATTGGCGGCACCGGGAAA
FOR-ZAPB(MICA)-50545	CAATTCAGGAGAGGAAAGTTGATGTGTTTAGAAGTGTTTG
REV-ZAPB(MICA)-50545	CAAACACTTCTAAACACATCAACTTTCCTCTCCTGAATTG
ZAPB-XBAI-REV	GATCTCTAGATTCGAGGACAACGGGTTGCTGTACGATCTG
ZAPB-SACI-FOR	GATCGAGCTCGCTACCCCCAGACCCCAGATGACGCTG
pBAD-E1	ACACTTTGCTATGCCATAGC
pBAD-E2	ACCGCTTCTGCGTTCTGATT
zapA-pBAD-FOR	ATCGGAATTCTCAATCAGCAGGAAGGTGGCATGTCTGCAC
zapA-pBAD-REV	TCGAGTCGACCCATAGTAAAGCGAAAAGTGTTATTCAAAG
RYHB-2FORBAMHI	ATCGGGATCCCTGCATAACGCTCGTTACACGGATTTGATT
RYHB-2REVSALI	TCGAGTCGACGAACGTACTGATCCATCCTCCGACTACCGC

Table S2. Predicted sRNAs with *zapB* mRNA as target and potential hybridization regions

sRNA	Energy [kcal/mol]	Nucleotide positions (sRNA)	Nucleotide positions (mRNA)
ryhB-2	-14.593	67-77	124-134
isrH	-14.177	348-379	347-378
ryhB-1	-13.432	51-61	124-134
isrA	-11.640	18-28	286-296
isrM	-11.510	254-260	290-296
csrC	-11.326	169-182	156-168
isrO	-11.296	99-171	229-313
cyaR	-10.538	24-35	137-148
isrJ	-10.340	23-42	318-339
csrB	-10.223	224-242	299-314
oxyS	-10.074	79-87	367-375
isrK	-10.041	40-70	301-331
isrG	-9.994	109-120	234-244
isrI	-9.602	133-149	70-88
micF	-9.591	30-59	89-120
rygD	-9.280	2-23	320-349
isrL	-9.144	99-109	205-215
invR	-9.113	3-38	78-115
isrF	-8.839	78-89	249-261
tpke	-8.733	227-238	142-153
micA	-8.708	4-13	52-61
gcvB	-8.685	135-144	214-223
rseX	-8.506	7-58	172-224
isrN	-8.502	7-25	234-254
isrQ	-8.479	68-80	143 -154
spf	-8.286	20-34	188-203
rprA	-8.267	46-69	230-253
